# Enhancement of the piezoelectric response of corona charged Ag/P(VDF-TrFE) nanocomposites for energy harvesting devices

**DOI:** 10.1038/s41598-026-46151-3

**Published:** 2026-04-21

**Authors:** A. Hassan, A. Habib, T. Fahmy, A. Magdy

**Affiliations:** 1 Polymer Research Group, Physics Department, Faculty of Science, Mansoura University, Mansoura, 35516 Egypt; 2 Faculty of Health Science Technology, Mansoura National University, New Mansoura, 35516 Egypt

**Keywords:** P(VDF-TrFE), TSDC, Corona poling, Electroactive β-phase, Piezoelectricity, Engineering, Materials science, Nanoscience and technology

## Abstract

Poly (vinylidene fluoride-co-trifluoroethylene)/Ag nanocomposites are fabricated in-situ using casting technique. Energy-Dispersive X-ray Spectroscopy (EDX), X-ray diffraction (XRD), Fourier transform infrared (FT-IR), Transmission electron microscopy (TEM) and UV-Vis spectroscopy are employed to investigate the structural and optical properties. Analysis of XRD measurements demonstrated that the crystallinity degree of P(VDF-TrFE) copolymer is enhanced upon increasing the AgNPs content. FTIR analysis showed that the electroactive β-phase is enhanced in the nanocomposite samples with increasing the content of AgNPs. UV-Vis results showed that both direct and indirect energy gaps (*E*_*dg*_*/E*_*ig*_) are reduced from (4.89/2.85) for pure P(VDF-TrFE) to (4.13/2.19) eV for P(VDF-TrFE)/0.28wt%Ag nanocomposite. Global TSDC measurements revealed that the ferroelectric-paraelectric phase transition is occurred at 323 K for pure P(VDF-TrFE) and decreased to 317 K after embedding AgNPs. The piezoelectric activity is found to enhance with increasing applied stress, measuring temperature and AgNPs content. The piezoelectric coefficient (d_33_) is optimized from 11.7 pC/N for pure P(VDF-TrFE) to 38.3 pC/N for P(VDF-TrFE)/0.28 wt%Ag nanocomposite at 6.24 × 10^5^ Pa. Our results provide prediction for the design of a novel flexible piezoelectric material capable of energy harvesting applications.

## Introduction

It is worth noting that with technological advancements and energy constraints, the sources of renewable energy have become one of the fastest growing areas of research. Piezoelectric materials have received significant attention over the past decade due to their flexibility, light weight, and ease of fabrication. Piezoelectric polymers offer numerous advantages due to their flexibility, low cost, long-term stability, and solution processing ability^1^. They are commonly used in sensors, actuators, converters and energy harvesting applications^2,3^. PVDF and its copolymer, polyvinylidene trifluoroethylene fluoride P(VDF-TrFE), have been the subject of extensive research into various piezoelectric materials^4^. PVDF is a polymorphic material with the formula (C_2_H_2_F_2_)_n_, often used for its ferroelectric and piezoelectric properties in specific chemical configurations. It has five different crystalline phases, called α, β, γ, ε, and δ phases^5^. The β and γ phases are electroactive, but the γ-phase is less electrically active than the β phase. On the other side, α-phase is nonpolar and is referred to as the non-electro active phase^6^.

Chemical addition of trifluoroethylene (TrFE) monomer to PVDF at a certain molar content is a simple and effective method to produce a highly polar β-phase. The phase transition behavior is significantly affected by the introduction of TrFE (-CF_2_-CFH-) groups into the PVDF matrix. By expanding the unit cell volume and the interplanar distance within the ferroelectric phase, the TrFE unit changes the crystal structure of pure PVDF^6^. Consequently, the majority of electrons are attracted to the fluorine side of the polymer chain, causing polarization^7^. Methods for improving the β-phase include adding fillers, mechanical stretching, electrical poling and using polar solvents for decomposition and recrystallization^8^. Corona polarization using very high voltage is a novel method for forming the β-phase of PVDF and enhances piezoelectricity with several properties useful for high-quality applications^9^. The improved electrical properties of P(VDF-TrFE)-based composite materials supplemented with metal nanoparticles have received significant attention recently.

Silver nanoparticles (AgNPs) are among the most widely used nanomaterials, because of their superior catalytic, electronic, optical, electrical, eco-friendly, relatively cheap and eco-friendly and antibacterial qualities^10,11^. It is worth noting that AgNPs and other noble metals have diverse applications in the field of catalysis^12^, sensors^13^, microelectronics^14^, biomedical^15^, energy harvesting^16^ and piezoelectric nanogenerator^17^. There are several methods for creating AgNPs, including sol-gel^18^, chemical reduction^19^, electrochemical^20^, green synthetic^21^, and in situ method^22^. The characteristics of nanoparticles, such as their size, shape, composition, and spatial distribution, are known to be influenced by their microstructure^23^. Since AgNPs can act as heterogeneous crystallization nuclei, they were fully induced to generate the piezoelectric β-phase and thus improve the piezoelectric behavior^24^.

Wu et al.. investigated the effect of silver nanowires (AgNWs) on the piezoelectric properties of poly(vinylidene fluoride-co-hexafluoropropylene) P(VDF-HFP). They found that the AgNWs act as nucleating agents in the β-phase transformation. The piezoelectric properties were also evaluated using output voltage and harvested energy density, and it was found that the harvested energy density increased by 159%^25^. Pike et al. investigated the effect of AgNPs on the structural and piezoelectric properties of P(VDF-TrFE) polymer. They found that the AgNPs influence the internal crystalline structure of the polymer chains, and the highest piezoelectric charge coefficient values ​​were also recorded with increasing silver nanoparticle content, reaching 20.23 pC/N^26^. Milenkovi´c et al. studied the effect of AgNPs incorporating on the piezoelectric activity of PVDF. Their results showed that incorporating silver nanoparticles into PVDF enhanced the formation of the β-phase crystalline fraction, thereby improving the piezoelectricity. The highest piezoelectricity coefficient was observed at a concentration of 0.3% silver nanoparticles^27^.

An efficient way to interpret relaxation features both numerically and qualitatively is to use the thermally stimulated depolarization current (TSDC) technique^28–31^. For decades, it has been widely used to describe the various relaxation mechanisms in polymeric materials^32,33^. TSDC theory has been widely applied to study various relaxation processes and molecular dynamics in ferroelectric polymers, despite it was initially designed to study the relaxation of thermally frozen dipoles^34,35^. The TSDC technique relies on applying a continuous polarized electric field at a specific temperature to align the polar groups within the polymer. The samples are then gradually cooled to room temperature while the electric field is continuously applied for freezing the dipoles. Then the applied electric field is turned off, and the samples are heated again. During this heating process, the polarized dipoles will relax, generating a depolarization current. This current is associated with the molecular motions and studies the internal shape and physical structure of material, giving important insights into molecular interactions^36,37^.

Since the piezoelectric activity of PVDF is much lower than that of piezoelectric ceramics, two techniques are used to improve the piezoelectric activity of PVDF and its copolymers: enhancing the residual polarization and increasing the piezoelectric β-phase content^38^. It is difficult to obtain a high polar β-phase content through conventional solution processing and melting^38^, but it is achieved through several strategies including heat treatment, mechanical stretching, polarization and the use of nanofillers^39^. The limited surface area between conductive fillers and P(VDF-TrFE) molecular chains poses a challenge for achieving dipole-filler interaction and thus increasing their polarization. The embedding of conductive fillers into the polymer matrix potentially increases the electric field intensity, leading to increased polarization of the polymer dipoles. Several active conductive nanofillers have been used for this purpose, such as silver nanoparticles (AgNPs)^40^, silver nanowires (AgNWs)^41^, graphene (rGO)^42^ and carbon nanotubes (CNTs)^43^. Silver is a unique material because of its relatively low cost and excellent electrical properties. Moreover, AgNPs have been employed as nanofiller in polymer matrix due to its excellent ability to enhance the β -phase structure of polyvinyl chloride (PVDF)^44^. By adding AgNPs to PVDF, β-phase content of about 80% can be obtained. The interactions between the electron-rich silver nanoparticles and the fluorine atoms will increase the polarization, thereby improving the piezoelectricity and the content of β-phase^44^.

In this work, the corona poling approach is applied to develop and improve the structure of novel piezoelectric nanocomposites of P(VDF-TrFE) and AgNPs that exhibit high piezoelectric efficiency. Using XRD and FTIR measurements, the phase change of the nanocomposites to improve the electroactive β-phase is investigated. Additionally, UV-Vis spectra are analyzed to calculate the direct and indirect bandgap energies of the different produced nanocomposites. Moreover, TGA and TSDC techniques are used to assess the thermal characteristics of nanocomposite films. A simple and efficient method for raising the piezoelectric coefficient of P(VDF-TrFE) doped with varying amounts of AgNPs is explained. The produced nanocomposite samples may open the door for optoelectronic devices and energy harvesting at the nano and microscales.

## Experimental work

### Materials

P(VDF-TrFE) with 65 mol% of VDF and 35 mol% of TrFE was purchased from Solvay (Belgium). Dimethyl formamide (DMF) was purchased from El Nasr pharmaceutical chemical company (Egypt). Silver nitrate (AgNO_3_), crystal, ACS, 99%, obtained from GAMMA laboratory chemicals, M.W = 169.87.

### Preparation method

#### Preparation of silver nanoparticles

In order to prepare silver nanoparticles, specific amount of AgNO_3_ crystals was dissolved in DMF solvent at room temperature (303 K) for 15 min. DMF solvent act as a reducing agent which produce a clear yellow solution that indicate the preparation of silver nanoparticles.

#### Preparation of Ag /P(VDF-TrFE) nanocomposite

**Fig. 1:**
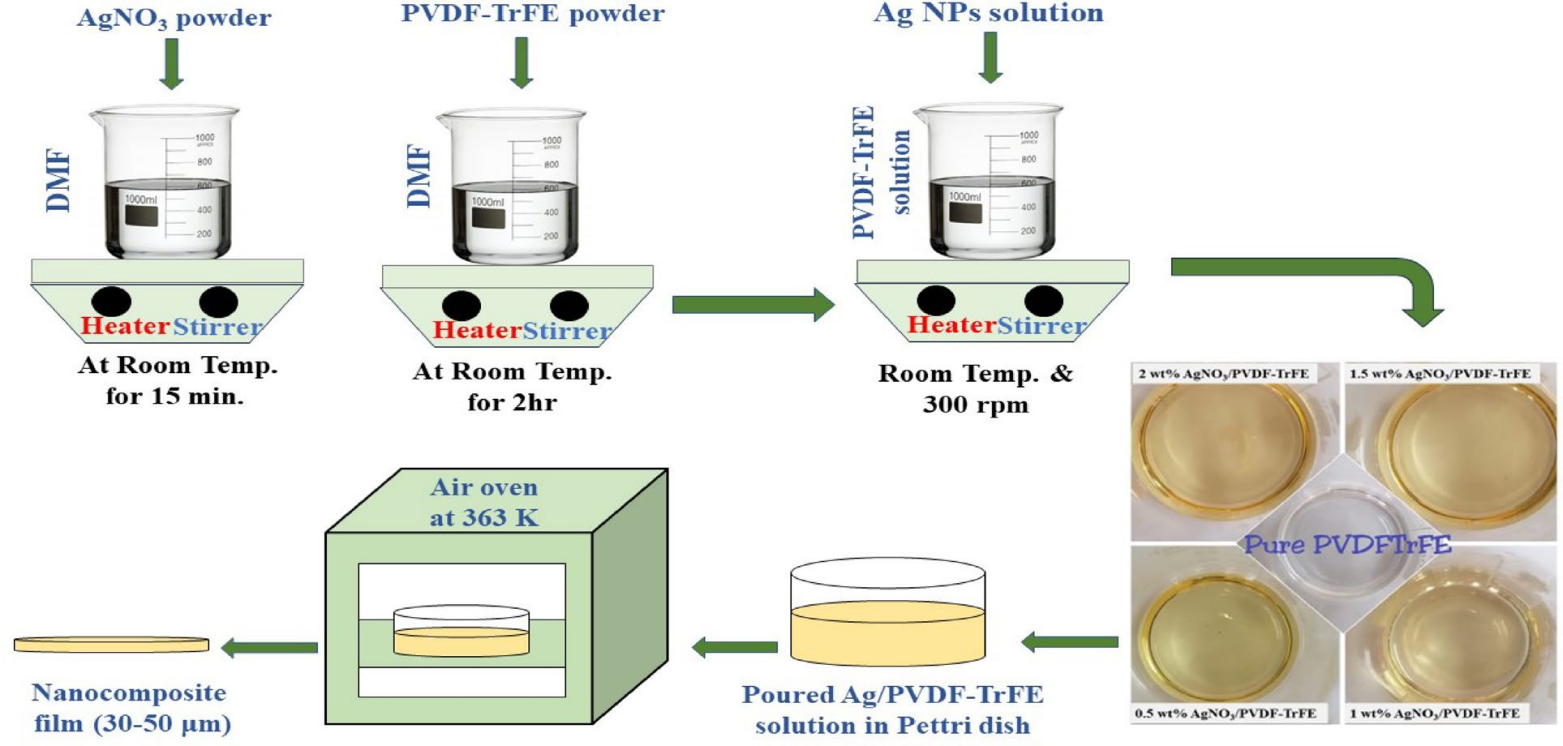
Scheme of preparation method of Ag/ P(VDF-TrFE) nanocomposites.

To prepare Ag/P(VDF-TrFE) nanocomposite, firstly, P(VDF-TrFE) copolymer powder was dissolved in DMF with magnetic stirring for 2 h at room temperature. Then, the earlier prepared silver nanoparticle solution with different contents is mixed with the prepared polymer solution. Next, the nanocomposite solutions were continuously stirred at room temperature for 2 h, followed by ultrasonicated for 30 min. Finally, the prepared nanocomposite solutions were dried into glass Petri dishes at 363 K in an oven for only one day to form nanocomposite films with approximately thickness of 30–50 μm, synthetic process is presented in Fig. 1.

### Measuring techniques

#### Characterization tools

Transmission electron microscope (TEM) technique (HRTEM, JEM 2100, JEOL) and EDX were achieved at the unit of electron microscope, Faculty of Agriculture, Mansoura University, Egypt. XRD measurements are measured using a Bruker D8 advance powder XRD with a CuK_α_ radiation source with wavelength of 1.5418 Ǻ at 40 kV and 50 mA with scan rate of 3°/min in the range from 5^0^ to 70^o^. FTIR spectra are performed by Thermo Scientific iD5 ATR in the range from 4000 to 400 cm^− 1^ with a spectral resolution of 1 cm^− 1^. UV-Vis spectra were achieved by ATI Unicam UV-Vis spectrophotometer (Mattson Co. UK) in the range 200–1100 nm.

#### Electrical measurement techniques

##### Thermally stimulated depolarization current TSDC setup

**Fig. 2:**
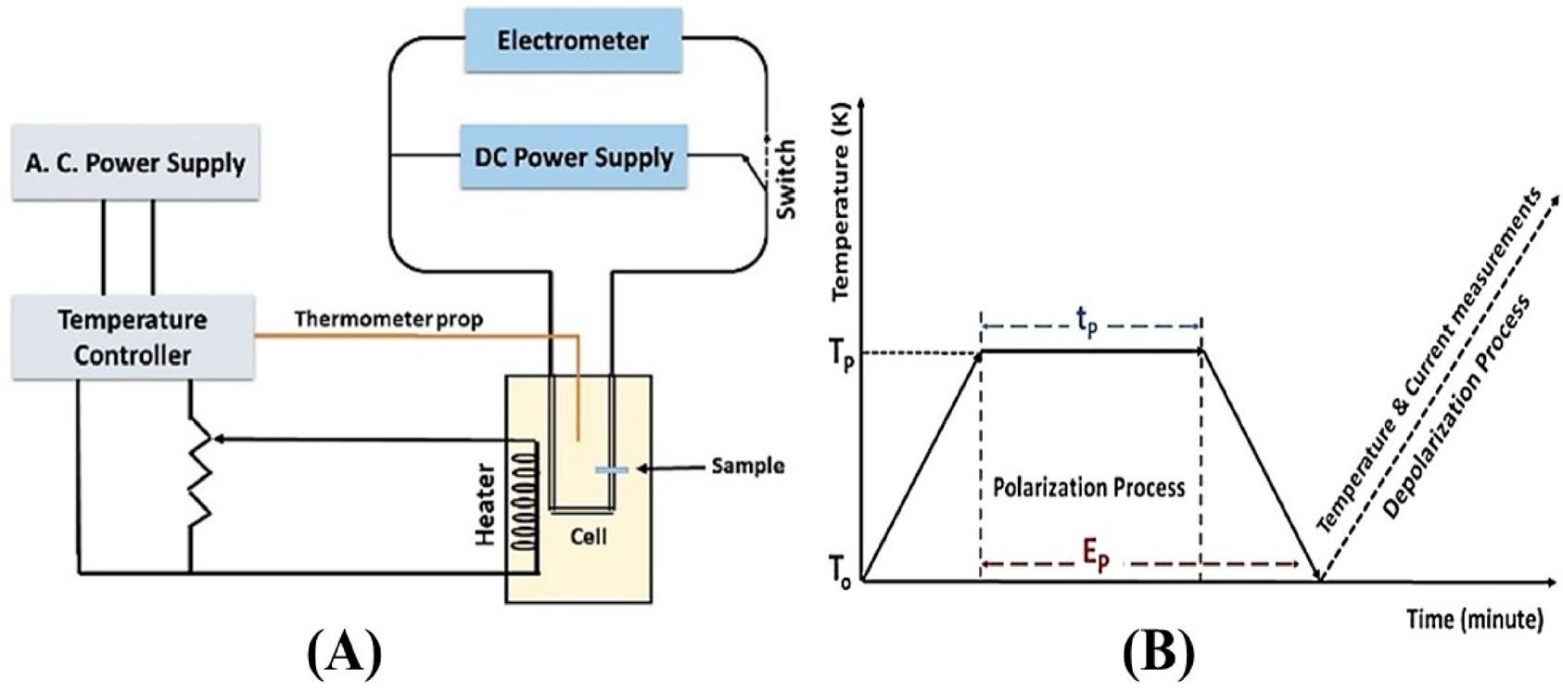
Schematic diagram of (**A**) the TSDC measurement system and (**B**) the TSDC experiment procedure.

At the first, the sample is coated by carbon electrode, then it was placed in an oven for heating to a specific temperature entitled the polarization temperature (T_p_) which can be chosen just less than the transition temperature of the sample. When the specific temperature of sample is achieved, a continuous electric field (E_p_) was applied for a certain time entitled polarization time (t_p_). After that, in the presence of the electric field, the sample is slowly cooled to room temperature. Then, the electric field is cut off, moreover the electrodes are short-circuited for 15 min to eliminate any anomalous charges^35^. Finally, the global TSDC spectra can be carried out by rewarming the samples at a constant heating rate (*b* = 3 K/min), as illustrated in Fig. 2.

##### Piezoelectric measurements

**Fig. 3:**
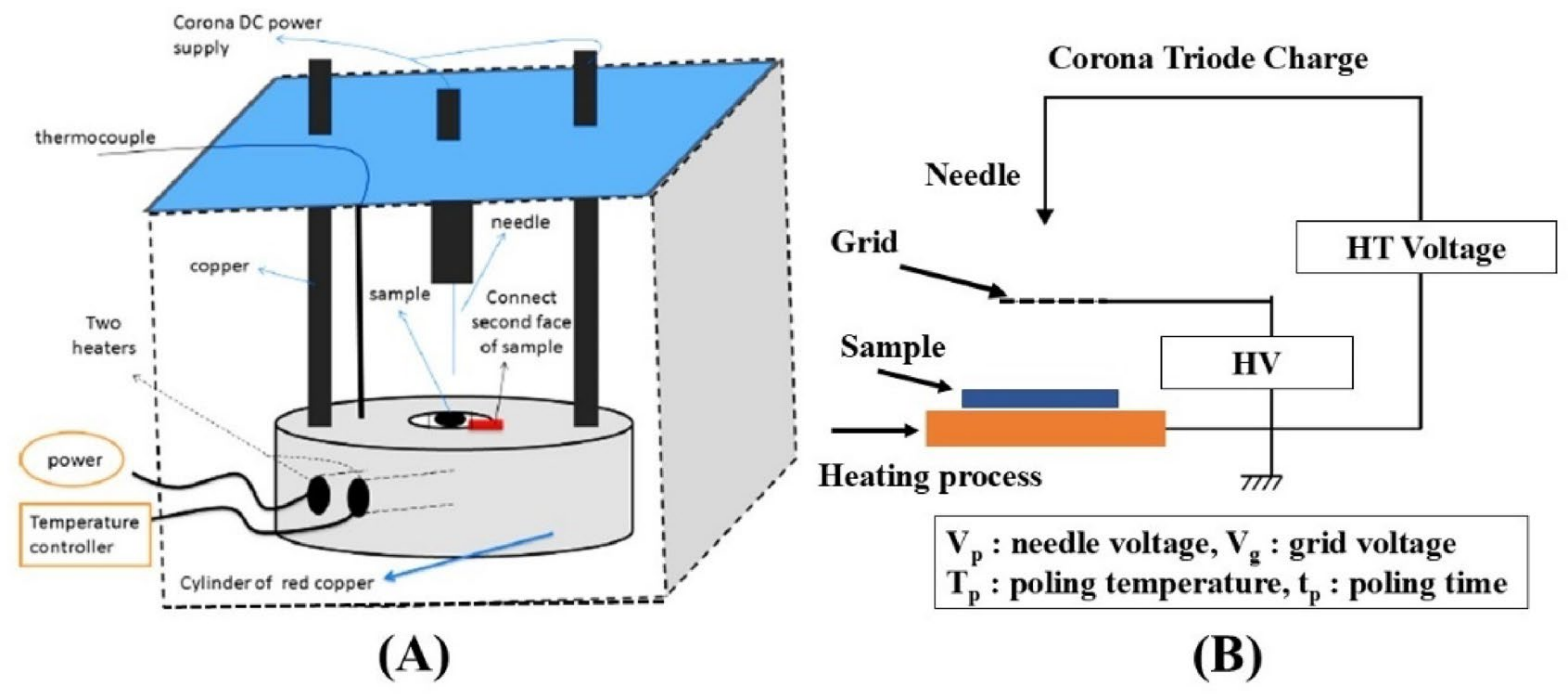
Sketch diagram (**A**) Cell used during the measurements and (**B**) Circuit used during corona charging.

The ex-prepared polymer films were polarized using a homemade setup^45^ using the triode corona charging technique. First, a high value of DC voltage was supplied to the cell’s needle which far with certain distance from the top surface of the sample. In contrast, the sample’s other surface was coated with carbon electrode and grounded. When the ionization capacity of the needle for the surrounding air was achieved, ionic species were formed and moved forward towards the ground terminal. This process resulted in the sample’s electric dipole polarization, as the charge density on the sample surface was appropriate plenty that the ions activated to move towards the other side of the sample. The prepared sample’s polarization approved at specific conditions of polarizing DC corona voltage, V_p_ = 8.5 kV and polarizing temperature T_p_= 363 K at constant polarizing time t_p_= 10 min. Next, polarized bipolar electrets were created and frozen by cooling the samples gradually to room temperature under a DC corona polarized electric field. The polarized electric field was then removed, and the prepared sample was short-circuited for 5 minutes to waste any stray surface charges. Then, the piezoelectric charge was recorded at different applied pressure from (2.5–6.24)x10^5^ Pa and various measuring temperature T_m_ from (323–363) K using Keithley 485 Picometer, as shown in Fig. 3.

## Results and discussion

### EDX

**Fig. 4:**
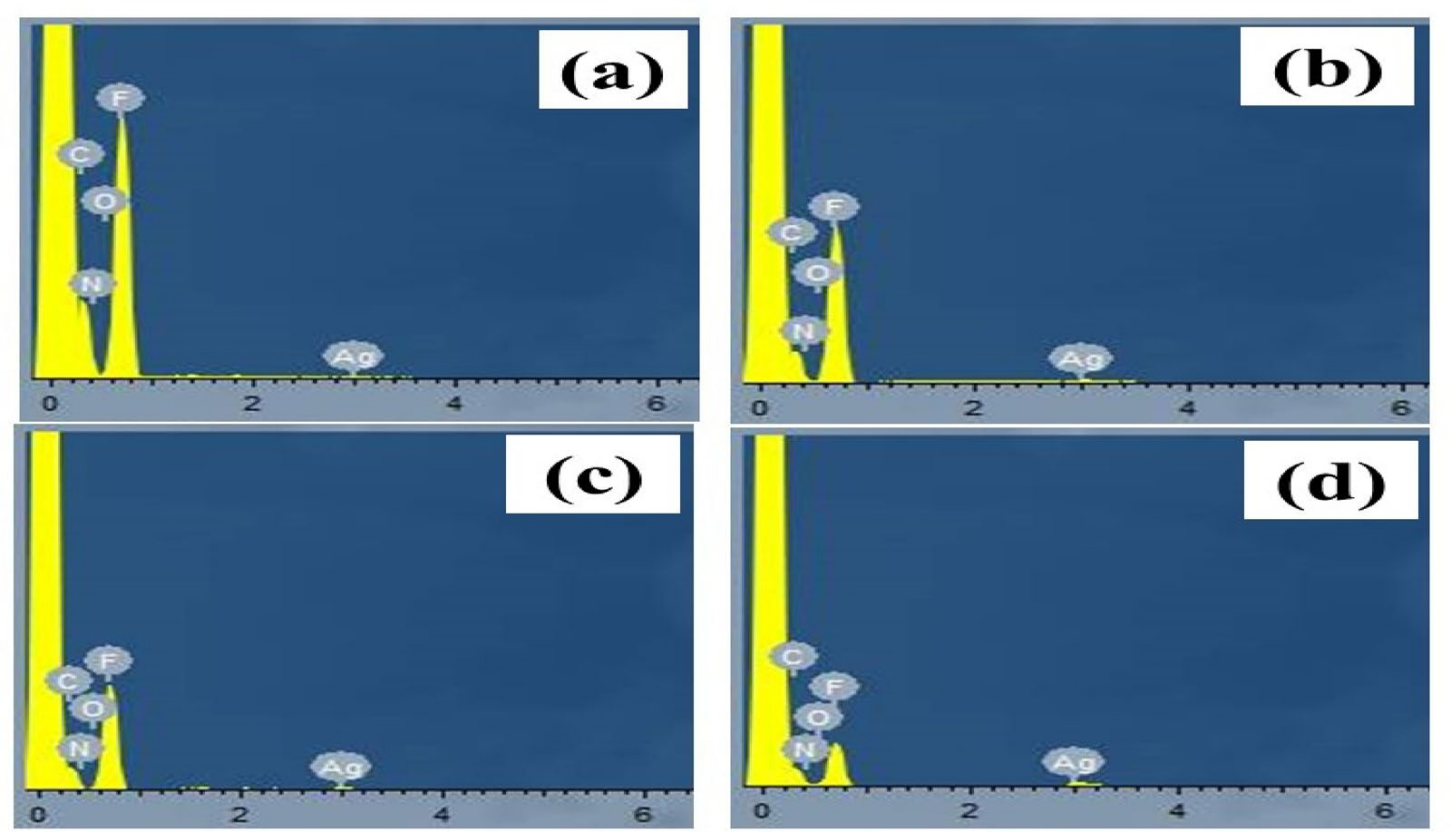
EDX of (**a**) 0.5% wt AgNO_3_/P(DVF-TrFE), (**b**) 1% wt AgNO_3_/P(VDF-TrFE), (**c**) 1.5% wt AgNO_3_/P(VDF-TrFE) and (**d**) 2% wt AgNO_3_/P(VDF-TrFE) nanocomposite.

The concentrations of AgNPs per weight in the polymer matrix were specified by using EDX, as shown in Fig. 4. It was found that the addition of AgNO_3_ to P(VDF-TrFE) polymer matrix with various concentrations 0.5, 1, 1.5 and 2 wt%, resulted in the introduction of various concentrations of AgNPs of 0.04, 0.13, 0.28 and 0.48 wt% into P(VDF-TrFE) matrix, respectively.

### XRD

**Fig. 5:**
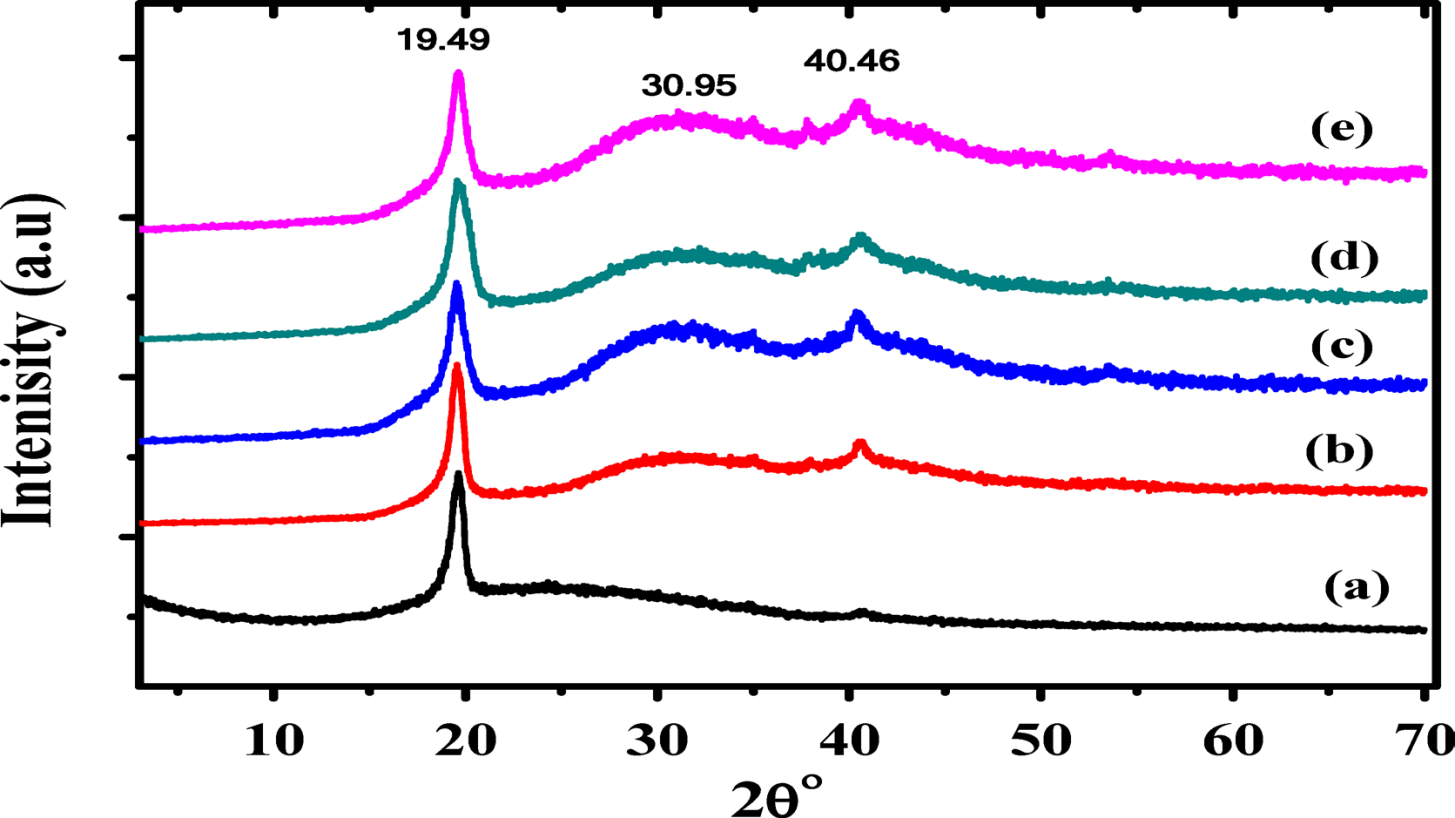
XRD patteren of (**a**) pure P(VDF-TrFE), (**b**) 0.04 wt%, (**c**) 0.13 wt%, (**d**) 0.28 wt% and (**e**) 0.48 wt% Ag/P(VDF-TrFE) nanocomposite. It is observed that the degree of crystalliity increased after introducing Ag nanoparticles to P(VDF-TrFE) matrix.

The structural parameters of P(VDF-TrFE)/Ag nanocomposite were estimated by using XRD. Figure 5 illustrated XRD patterns of P(VDF-TrFE) and P(VDF-TrFE)/Ag nanocomposite samples. The diffraction peak at 2θ = 19.49° of β-phase in P(VDF-TrFE) with (110)/(200) planes of all-trans conformation (β phase) was observed, as shown in Fig. 5a, confirming the semicrystalline nature of P(VDF-TrFE)^46^. Upon doping with AgNPs a slight change in the position and intensity of β-phase diffraction peaks in highly doped sample of P(VDF-TrFE)/Ag nanocomposites was observed, as shown in Fig. 5e. A broad peak at 30.95^o^ was appeared related to (111) crystal planes of AgNPs^47^ and another small sharp peak at 40.46◦ was appeared and attributed to the (201)/(111) planes and is consistent with β phase crystals, indicating the presence of the ferroelectric β phase^48^, confirming the effect of AgNPs in the composite samples. The change in the intensity and position of the peaks after addition AgNPs is attributed to the interactions between the CH_2_/CF_2_ dipoles of P(VDF-TrFE) and AgNPs at the interfaces, leading to the generation of larger quantities of β-phase in the nanocomposite samples. This means that β-phase fraction in the P(VDF-TrFE)/Ag nanocomposite samples is improved, indicating that the piezoelectric response of these nanocomposites will be enhanced^49^. The crystal size (*D*) and lattice strain (ε) are computed for the investigated samples using Debye-Scherrer equation as follows^50^:

1 $$\:D=\frac{0.9\lambda\:}{\beta\:{cos}\theta\:}$$

2 $$\varepsilon = \frac{\beta }{{4\tan \theta }}$$

Various another structural parameters such as number of crystallites per unit area (*N*_*c*_) and the density of dislocation (δ) are calculated as follows^51^:

3 $$\:{N}_{c}=\frac{d}{{D}^{3}}$$

4 $$\:\delta\:=\frac{1}{{D}^{2}}$$

where *d* is thickness of the sample. Structural parameters are calculated and listed in Table 1.

**Table 1:** The parameters of the structure of PVDF-TrFE and Ag/PVDF-TrFE nanocomposites.

Structural parameters		Pure PVDF-TrFE	0.04 wt % Ag/PVDF-TrFE	0.13 wt % Ag/PVDF-TrFE	0.28 wt% Ag/PVDF-TrFE	0.48 wt% Ag/PVDF-TrFE
D (nm)		11.42	10.89	8.96	7.75	10.21
ε (10^− 2^)		1.78	1.87	2.27	2.62	1.99
N_c_ (nm^− 2^)		13.41	15.47	27.82	42.92	18.81
δ (10^− 3^ nm^− 2^)		7.66	8.42	12.46	16.63	9.59

### FTIR

**Fig. 6:**
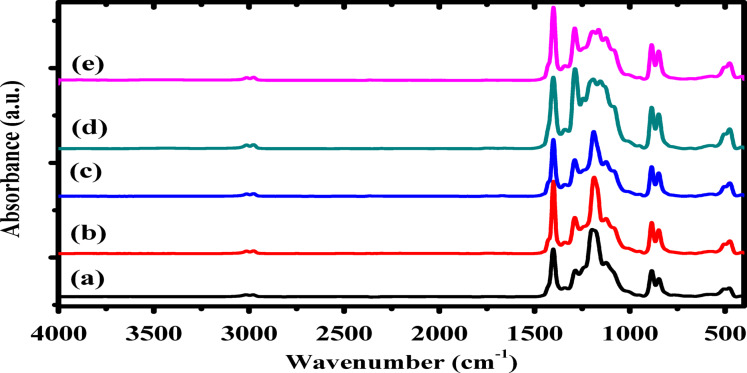
FTIR spectra of (**a**) pure P(VDF-TrFE), (**b**) 0.04 wt%, (**c**) 0.13 wt%, (**d**) 0.28 wt% and (**e**) 0.48 wt% Ag/P(VDF-TrFE) nanocomposite.

FTIR spectra of P(VDF-TrFE) and Ag/P(VDF-TrFE) nanocomposites with various concentrations of AgNPs in the region 1600 –400 cm^− 1^ were demonstrated in Fig. 6. FTIR spectrum of P(VDF-TrFE) showed many absorption bands such as 1402, 1288, 1184, 1121, 1078, 884, 848, 508 and 474 cm^− 1^. Band at 1402 cm^− 1^ is related to the combination between α, β and γ phases^52^. The absorption bands at 1288, 1121, 884, 848 and 508 cm^− 1^ are assigned to β-phase. These vibration bands confirmed the existence of ferroelectric β-phase in the P(VDF-TrFE) copolymer with all-trans chain conformation^53^. Band at 1288 cm^− 1^ is attributed to CF_2_ symmetric stretching coupled with the symmetric vibrations of C-C and the bending vibrations of C-C-C^24,25^. The absorbance bands at 884 and 848 cm^− 1^ are assigned to CF_2_ asymmetric stretching or CH_2_ rocking and to CF_2_ symmetric stretching, whereas the band at 507 cm^− 1^ is related to CF_2_ wagging and bending. The band of absorption at 1078 cm^− 1^ is ascribed to combination of β and γ phases^54^. Bands of absorption at 1184 and 474 cm^− 1^ are ascribed to α-phase^46^. However, different changes at the peak’s positions and intensities of 1288, 1184 and 1121 cm^− 1^, appeared indicating that the inclusion of silver nanoparticles has a significant role on the crystalline transformation of pure P(VDF-TrFE). The intensity of peak at 1184 cm^− 1^ is decreased and shifted to higher wavenumber. On the other hand, the peak’s intensities at 1288 and 1121 cm^− 1^ are increased with a slight shift to lower wavenumber. The variation of these peaks indicates that the α-phase has transformed into the β-phase^26,27^, since by adding silver nanoparticles to P(VDF-TrFE), the partially charged fluorine atoms, because of their strong electron-withdrawing property, will be attracted to the silver nanofiller surface, resulting in a change in its configuration conformation^55^.

The electroactive β-phase fraction *F*(*β*) in the nanocomposites is computed as follows^56^:

5 $$\:F\left(\beta\:\right)=\frac{{A}_{\beta\:}}{{(K}_{\beta\:}/{K}_{{\upalpha\:}}){A}_{{\upalpha\:}}+{A}_{\beta\:}}$$

where *A*_α_ is the intensity of absorption peak of α-phase at 774 cm^− 1^ and *A*_β_ is the intensity of β*−*phase at 848 cm^− 1^. Also, the absorption coefficients *K*_β_ and *K*_α_ of these respective bands and equal 7.7 × 10^4^ and 6.1 × 10^4^ cm^2^ mol^− 1^, respectively^57^. It was found that, the electroactive β-phase fraction is enhanced and increased from 87.21% for pure P(VDF-TrFE) to 92.84% for 0.28wt% P(VDF-TrFE)/Ag nanocomposite sample as listed in Table 2. In general, fillers play a vital role in the polymer crystallization process by first acting as nucleation sites for polymer crystallization, and second, by stabilizing the polymer chains. These two effects are opposing, as crystallization is accelerated by fillers, while chain stabilization delays the crystallization process. Our results are entirely consistent with what has been published previously. Therefore, with increasing filler content, the proportion of crystallized β-phase is expected to decrease. Issa et al. showed that the maximum value of β-phase crystallization is at 0.4 wt% of AgNPs in PVDF, and also Milenkovi´c et al. published that the maximum value of phase b crystallization is at 0.4 wt% of silver nanoparticles in polyvinyl chloride (PVDF) and then decreases thereafter with increasing silver nanoparticle content^27,44^.

**Table 2:** Electroactive β phase fraction of Ag/P(VDF-TrFE) nanocomposites.

Sample (wt%)	Pure P(VDF-TrFE)	0.04% Ag/ P(VDF-TrFE)	0.13% Ag/ P(VDF-TrFE)	0.28% Ag/ P(VDF-TrFE)	0.48% Ag/ P(VDF-TrFE)
β− fraction %	87.22	90.78	92.67	92.84	88.9

**Fig. 7:**
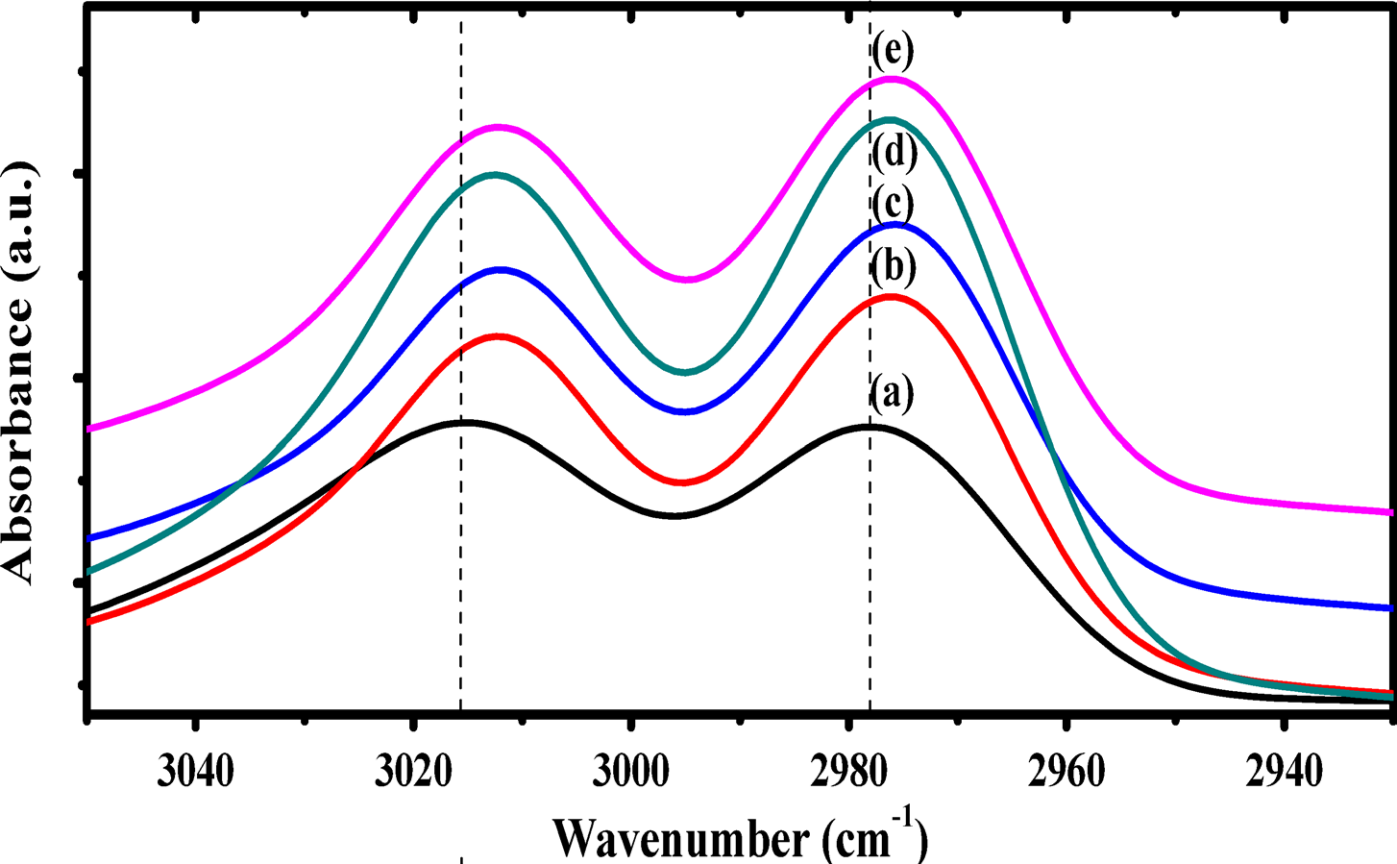
FTIR spectra of (**a**) pure P(VDF-TrFE), (**b**) 0.04 wt%, (**c**) 0.13 wt%, (**d**) 0.28 wt% and (**e**) 0.48 wt% Ag/P(VDF-TrFE) nanocomposite.

The gradual improvement in β-phase content after doping with silver nanoparticles may be attributed to the electrostatic interactions between the nano-filler and P(VDF-TrFE) chain^58^. These interactions will promote the zigzag (TTTT) formation of the P(VDF-TrFE) chain and hence the β−phase content will improve. The improvement of β-phase content due to the interfacial interactions between AgNPs and –CH_2_ dipoles could be confirmed by examining the bands in the range 3020 –2900 cm^− 1^, as the stretching vibrations of –CH_2_ groups are responsible for the existence of these bands. Also, the shift of –CH_2_ bands towards lower wavenumber already indicates the gradual improvement of the interfacial interactions in the Ag/P(VDF-TrFE) nanocomposites, as illustrated in Fig. 7^35^.

### TEM

**Fig. 8:**
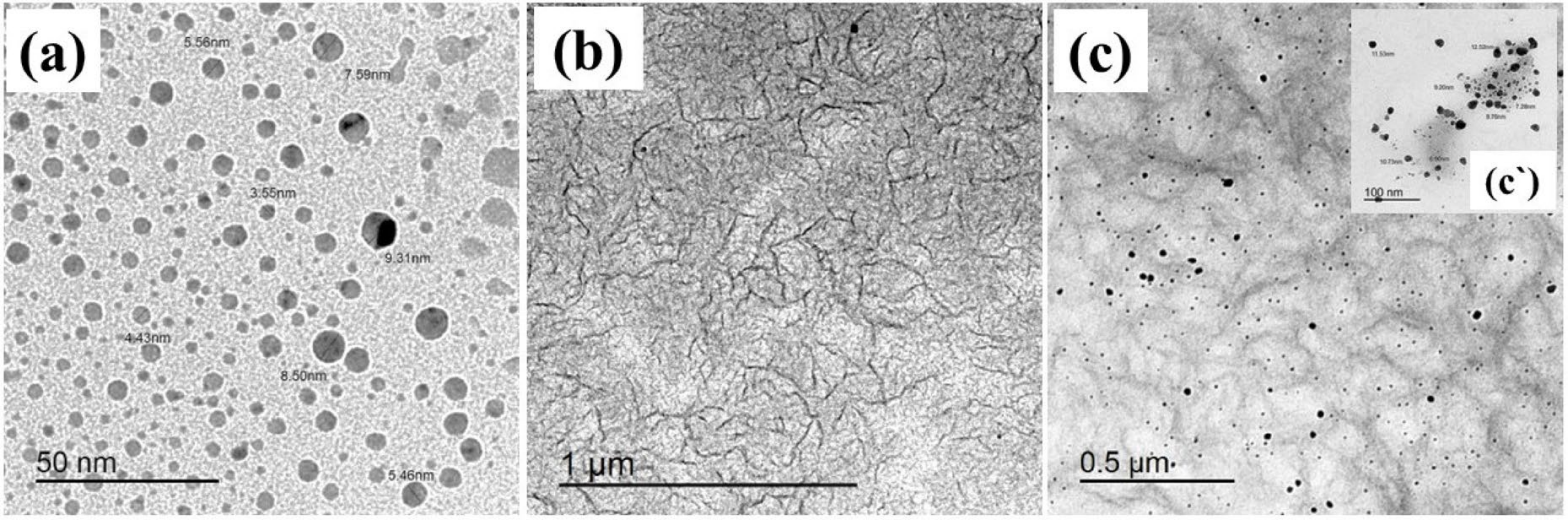
TEM patteren of (**a**) AgNPs, (**b**) P(VDF-TrFE) and (**c**) 0.13% wt Ag/P(VDF-TrFE) nanocomposite.

**Fig. 9:**
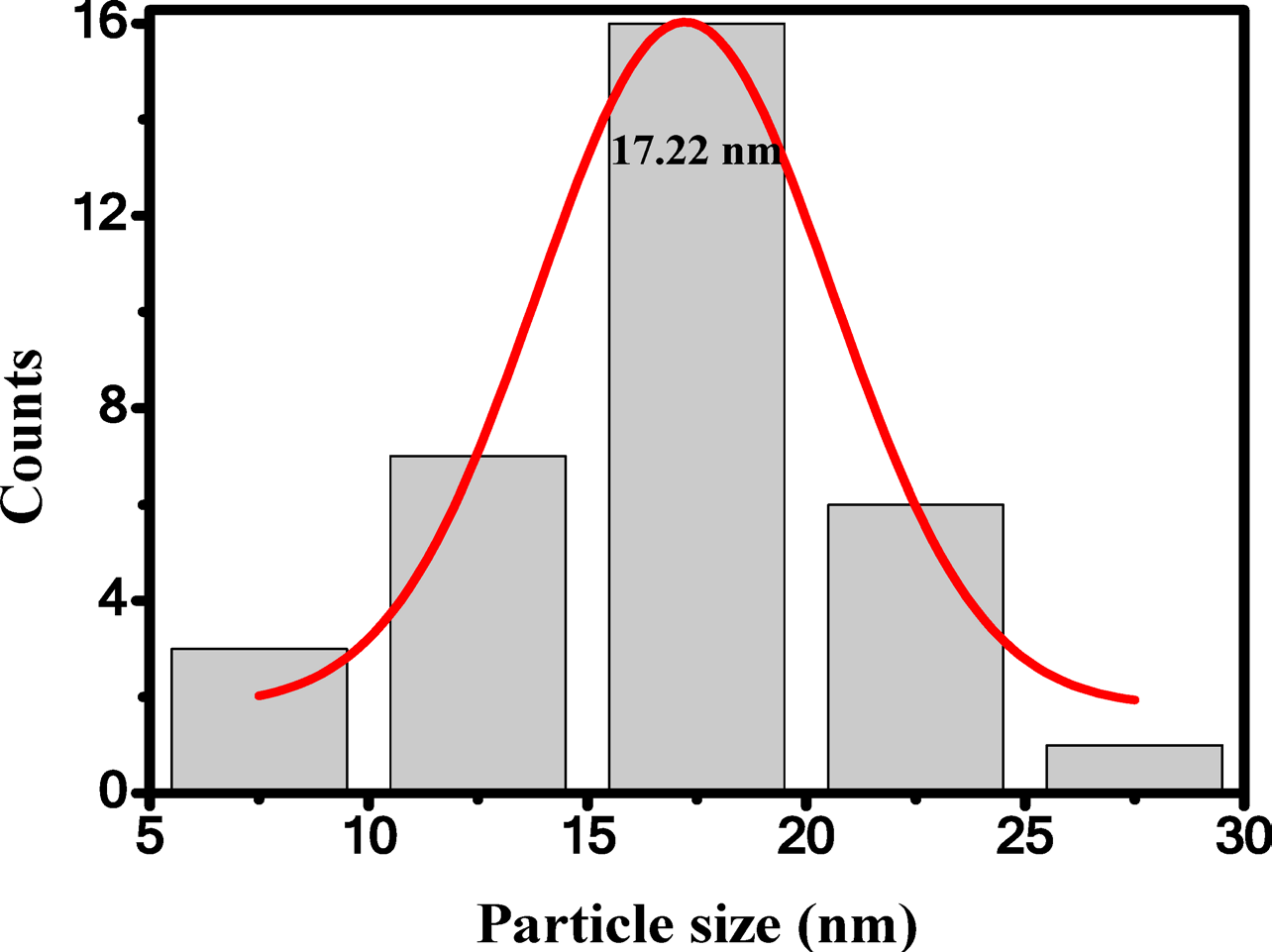
The average particle size of AgNPs in 0.13% wt Ag/P(VDF-TrFE) nanocomposite sample.

The morphology of Ag/P(VDF-TrFE) nanocomposite was studied using TEM technique as represented in Fig. 8. Pure Ag nanoparticles’s images showed a spherical shape, as shown in Fig. 8a, while pure P(VDF-TrFE) shows dendritic like structure, as shown in Fig. 8b. Figure 8c exhibited a homogeneous distribution of AgNPs within the P(VDF-TrFE) matrix. As shown in Fig. 8c, the incorporation of AgNPs in the matrix of P(VDF-TrFE) results in some morphological changes. Large amounts of silver nanoparticles appear to be aggregated on the surface of P(VDF-TrFE). These aggregations are attributed to the polymer-filler complexes formation through interactions with filler charges, leading to their irregular distribution in the P(VDF-TrFE) matrix^59,60^. It is also worth noting that in the modified P(VDF-TrFE), its stability can be enhanced by van der Waals or dipole-dipole interaction between CF_2_/CH_2_ and negative surface charges of the silver nanoparticles. These interactions would generate new forms and subsequently change the morphology of P(VDF-TrFE), which in turn significantly reduces the characteristic texture of α-phase. This implies that silver nanoparticles are able to inhibit the growth of the electrically inactive α-phase and thus enhance the electro-active phase formation, i.e., β-phase^61^. Therefore, the microstructure enhancement of P(VDF-TrFE) polymer resulting from the introduction of silver nanoparticles is expected to play a significant effect in improving the piezoelectric response and energy harvesting performance of nanocomposite samples. The particle size of AgNPs in the nanocomposite sample is estimated and it is found that the average value is around 17.22 nm, as illustrated in Fig. 9.

### UV-Vis spectra

**Fig. 10:**
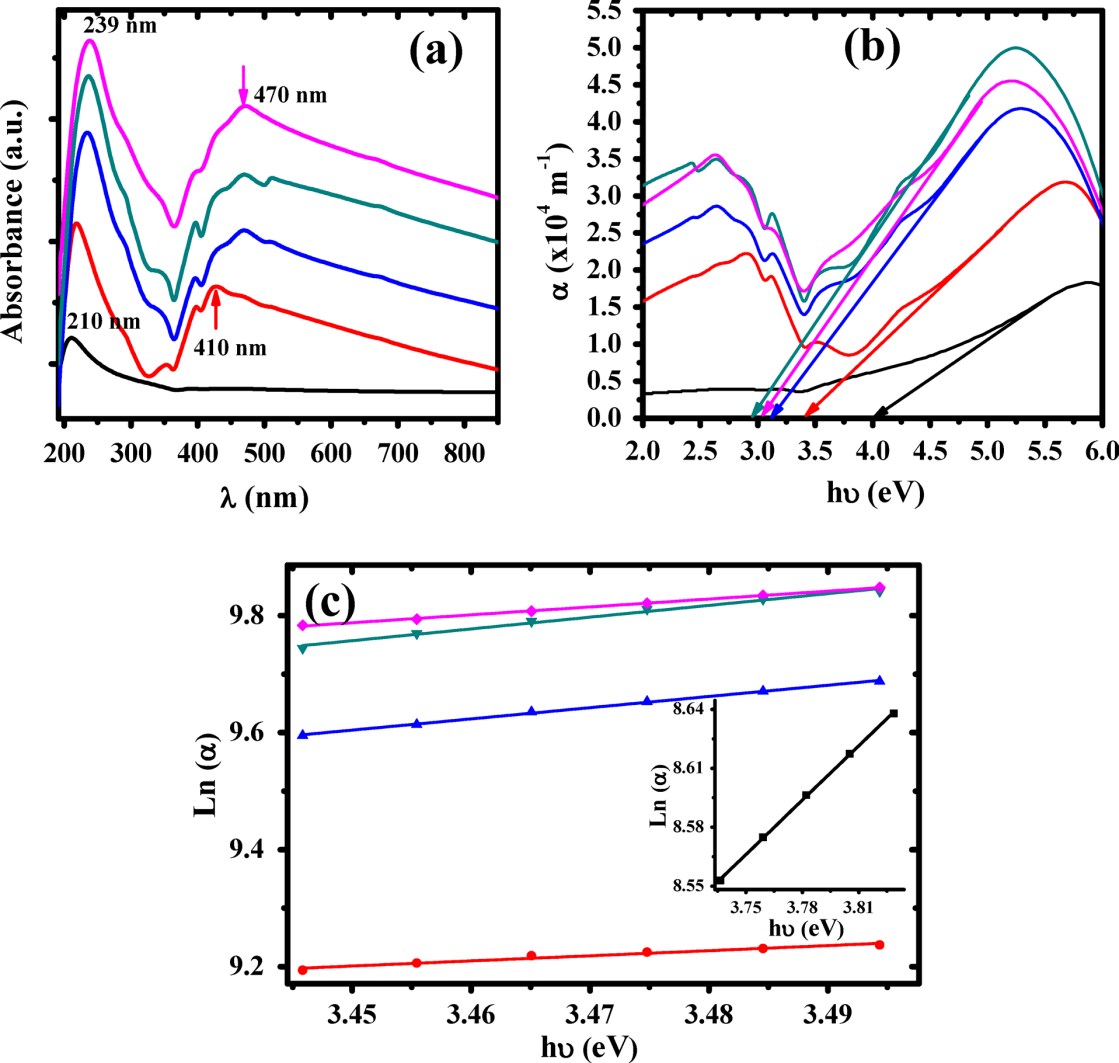
(**a**) The absorption versus λ, (**b**) α against hυ and (**c**) Ln α versus hυ for () P(VDF-TrFE), () 0.04 wt% Ag/P(VDF-TrFE), () 0.13 wt % Ag/P(VDF-TrFE), () 0.28 wt % Ag/P(VDF-TrFE) and () 0.48 wt % Ag/P(VDF-TrFE) NCs.

The optical properties of the composite samples with different content of AgNPs are investigated using UV–vis spectra. Figure 10a illustrated the absorption spectra of P(VDF-TrFE) and Ag/P(VDF-TrFE) nanocomposites in the range from 200 nm to 1100 nm. The spectrum of P(VDF-TrFE) showed an absorption peak at 210 nm and shifted to 239 nm with increasing the content of Ag nanoparticles. These bands were attributed to the π-π^*^ transition and the sharp absorption edge at 210 nm indicated the semicrystalline nature of P(VDF-TrFE)^62^. The spectrum of Ag/P(VDF-TrFE)/ nanocomposites displayed an absorption peak at 410 nm and shifted to 470 nm with increasing the content of AgNPs. This absorption peak is related to Ag nanoparticles^63^. Furthermore, it was observed that the absorbance of the nanocomposite samples changed and increased smoothly with increasing Ag doping content because the combined filling of doping ions with P(VDF-TrFE) chains absorb the incident radiation by free electrons at the shortest wavelengths. In general, AgNPs were homogeneously dispersed in P(VDF-TrFE) matrix and higher AgNPs content led to stronger absorption peaks. Figure 10b displays the absorption coefficient α (α=2.303 *A/d*, where *A* and *d* is the absorbance and sample thickness) variation of all samples versus the photon energy (hυ). The absorption edge was found to shift towards higher wavelength/lower energy and reduced from 4.01 eV for P(VDF-TrFE) to 3.01 for 0.28 wt% Ag/P(VDF-TrFE) nanocomposite sample. This shift in the absorption edge to higher wavelength reflects the change of the optical energy bandgap, which may be due to modification in the crystal phase of P(VDF-TrFE) matrix. The values of the absorption edge of all samples were given in Table 3.

**Table 3:** Optical parameters of PVDF-TrFE and Ag/PVDF-TrFE nanocomposite.

Structural parameters	Pure PVDF-TrFE	0.04 wt % Ag/PVDF-TrFE	0.13wt % Ag/PVDF-TrFE	0.28 wt% Ag/PVDF-TrFE	0.48 wt% Ag/PVDF-TrFE
E_ed_ (eV)	4.01	3.43	3.14	2.94	3.01
E_U_ (eV)	1.48	1.32	1.22	1.05	1.55
E_idg_ (eV)	2.85	2.63	2.38	2.19	2.25
E_dg_ (eV)	4.89	4.56	4.25	4.13	4.18

The absorption coefficient (α) displays an exponential dependence on the photon energy near the absorption edge and this correlation was defined as Urbach rule. The Urbach energy (*E*_*U*_) is computed as follows^64^:

6 $$\alpha = \alpha 0\,\exp \left( {\frac{{h\upsilon }}{{E_{U} }}} \right)$$

where α_0_ is a pre-exponential factor. Urbach energy values would provide more details about the optical behavior of Ag/P(VDF-TrFE) nanocomposite samples. Figure 10c depicts the dependence of ln (α) on the photon energy (*hυ*). Values of Urbach energy were calculated using the slope of the fitted straight lines and given in Table 3. It was found that the *E*_*U*_ values of the nanocomposites decreased from 1.48 eV for pure P(VDF-TrFE) to 1.05 eV for 0.28wt% Ag/P(VDF-TrFE) nanocomposite. This decreased in Urbach energy values is indicative to the structure modification of P(VDF-TrFE) matrix, which in turn would lead to decrease in the disorder of the nanocomposites which agree with both XRD and FTIR data.

**Fig. 11:**
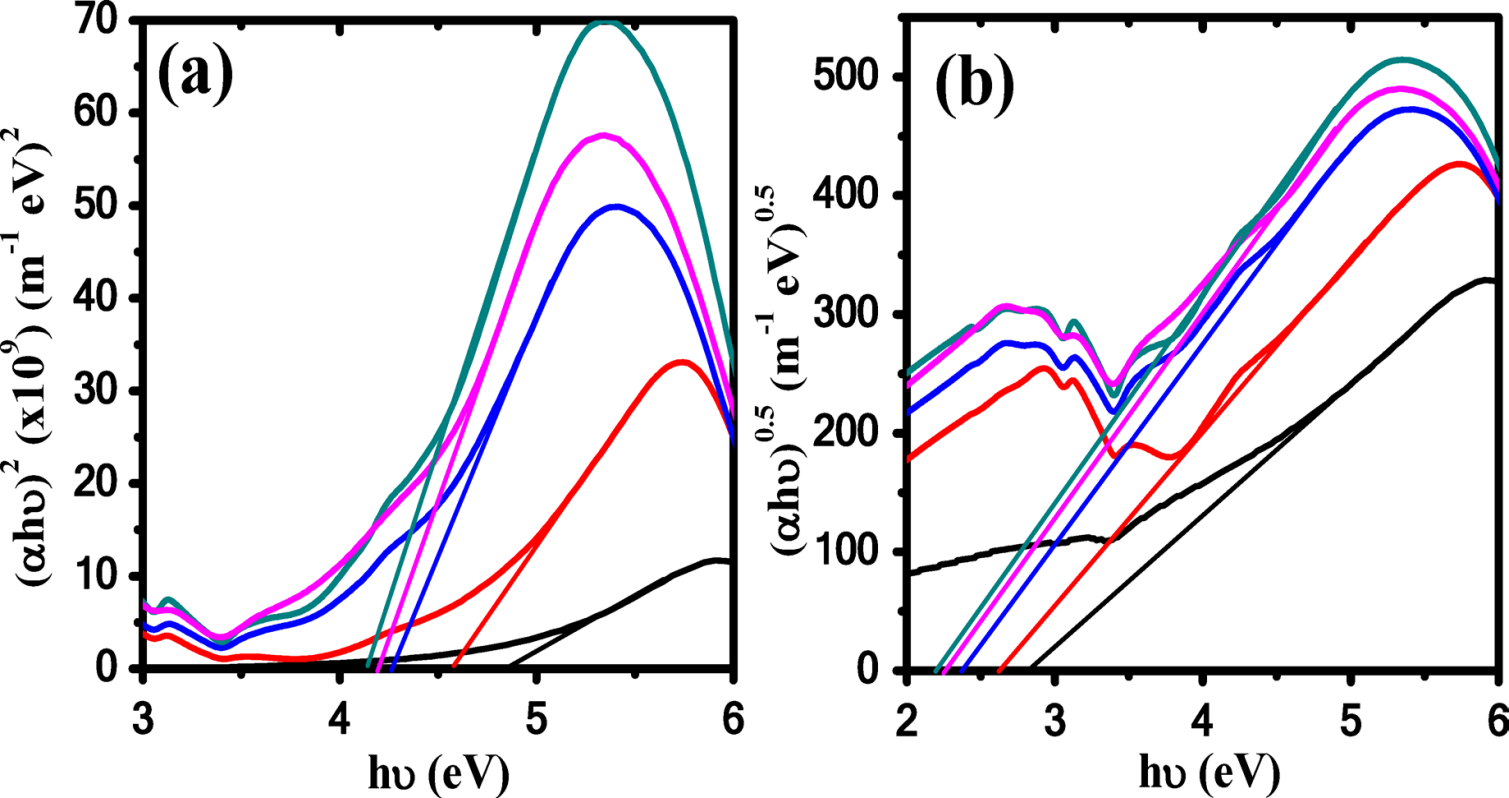
(**a**) (αhυ)^2^ vs. hυ and (**b**) (αhυ)^0.5^ vs. hυ. () P(VDF-TrFE), () 0.04 wt% Ag/P(VDF-TrFE), () 0.13 wt % Ag/P(VDF-TrFE), () 0.28 wt % Ag/P(VDF-TrFE) and () 0.48 wt % Ag/P(VDF-TrFE) NCs. .

The optical transitions nature in Ag/P(VDF-TrFE) nanocomposites was investigated using Tauc’s equation as follow^65^:

7 $$\alpha h\upsilon = B(h\upsilon - E_{g} )^{m}$$

where *B* is a constant and *m* is an exponent describes the electronic transitions nature, i.e., for the direct allowed transition (*m =* 1/2) and indirect allowed transition (*m =* 2), respectively. Figure 11 illustrates the dependence of (αhυ)^0.5^ and (αhυ)^2^ on hυ for all samples. Indirect (*E*_*ig*_) and direct (*E*_*dg*_) optical bandgap energies are calculated by extrapolate the linear portions of the curves of Fig. 11 to (αhυ)^0.5^ = 0 and (αhυ)^2^= 0 on the x-axis and given in Table 3. It was noted that both the indirect and direct energies were decreased with increasing content of AgNPs, which attributed to the new localized states near the band edge, which in turn reduces the Fermi level and hence affected the energy gap. Indirect and direct energies were reduced from 2.85 eV and 4.89 eV for pure P(VDF-TrFE) to 2.19 eV and 4.13 eV for 0.28 wt% Ag/P(VDF-TrFE), respectively. Our results of E_g_ are consistent with previously published data. Indulea et al. reported that the (*E*_*ig*_ /*E*_*dg*_) values are decreased from (4.96/5.66) eV for pure PVDF to (3.35/4.95) eV as ZnONPs content increases to 9 wt% and Hassan et al. noted that as the ZnONP content increases, the values of (*E*_*ig*_/*E*_*dg*_) are decreased from (2.89/4.82) eV for pure P(VDF-TrFE) to (1.69/3.85) eV to P(VDF-TrFE)/1.25 wt% ZnONPs^66,67^ It was also reported that with increasing Fe_2_O_3_ loading in P(VDF-TrFE), the (*E*_*ig*_/*E*_*dg*_) values ​​decrease from (3.70/4.65) eV to (2.78/3.5) eV^68^. The decrease in the bandgap is attributed to the modification of the structure based on the formation of new localized states in the region of energy gap after doping with silver nanoparticles^69^. The decrease in the optical energy values is related to the defect’s formation and thus the optical behavior of the material will be affected^70,71^.

### TSDC

**Fig. 12:**
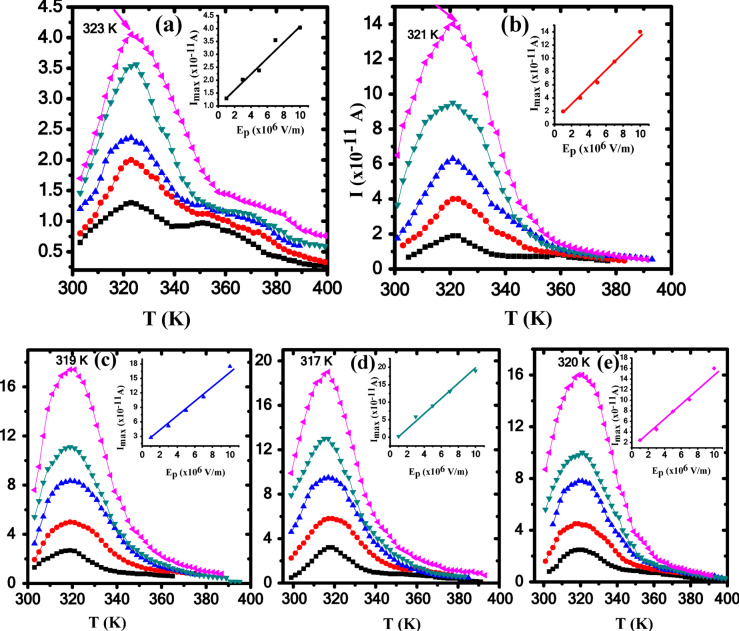
TSDC of (**a**) pure P(VDF-TrFE), (**b**) 0.04 wt%, (**c**) 0.13 wt%, (**d**) 0.28 wt% and (**e**) 0.48 wt% Ag/P(VDF-TrFE) nanocomposite. All samples were polarized at different polarizing electric field at different values of E_p_. )1 × 10^6^ V/m )3 × 10^6^ V/m )5 × 10^6^ V/m )7 × 10^6^ V/m ) 1 × 10^7^ V/m, fixed polarization temperature 353 K and time of 10 min.

TSDC was used in this work to investigate the behavior of relaxation modes of Ag/P(VDF-TrFE) nanocomposites and to estimate the molecular parameters such as the activation energy (*E*_*a*_) and relaxation time (τ_o_). Global TSDC spectra of pure P(VDF-TrFE) and Ag/P(VDF-TrFE) were represented in Fig. 12. As already well known P(VDF-TrFE) is a semi-crystalline copolymer containing crystalline lamellae within an amorphous matrix^72^. Figure 12a shows the TSDC spectrum of P(VDF-TrFE) in the temperature range 300–400 K under a variable polarization electric field. It is found that, this spectrum is characterized by a relaxation peak centered at 323 K. It is known that, electret fluoropolymers such as PVDF and its copolymers contain localized, irregularly distributed space charges that are injected around the ferroelectric crystalline regions and trapped in an irregular manner during the polarization process^73^. Therefore, we expect significant contributions from these trapped charges, in addition to the generated current by the dipole motion. Hence, the global TSDC spectrum of P(VDF-TrFE) is mainly caused by dipoles during the heating process, and presence of TSDC relaxation peak in the spectrum expresses the phase transition, which indicates that the molecular structure of P(VDF-TrFE) polymer has changed^74^. During the transition from ferroelectric to paraelectric phase, the depolarization current reaches its maximum, as shown in the Fig. 12. Also, the shoulder that appeared in the temperature range of 352 to 374 K is attributed to the release of charge carriers trapped in the ideal paraelectric phase structure^75^. This type of relaxation in the high temperature region is called space charge relaxation, i.e., ρ-relaxation^31,35^.

To differentiate between the different relaxations, the maximum current (I_m_) should be investigated versus the applied electric field (E_p_), as shown in the inset of Fig. 12a. TSDC peak current (I_m_) was found to increase linearly with increasing (E_p_). Moreover, no shift in the maximum peak position (T_m_) was observed, confirming that the main contribution to TSDC peak is attributed to the motion of the permanent C-C dipoles rather than induced dipoles^76^. This type of relaxation is mainly associated with the glass transition temperature (T_g_) of the polymer and is called dipolar relaxation, i.e., α-relaxation. On the other side, global TSDC spectra of Ag/P(VDF-TrFE) nanocomposites is shown in Fig. 12b–e. Generally, single mode of relaxation is observed for all nanocomposite samples. The TSDC position of the main peak is shifted to lower temperatures with the addition of AgNPs into P(VDF-TrFE) copolymer up to 0.28% AgNPs content.

**Fig. 13:**
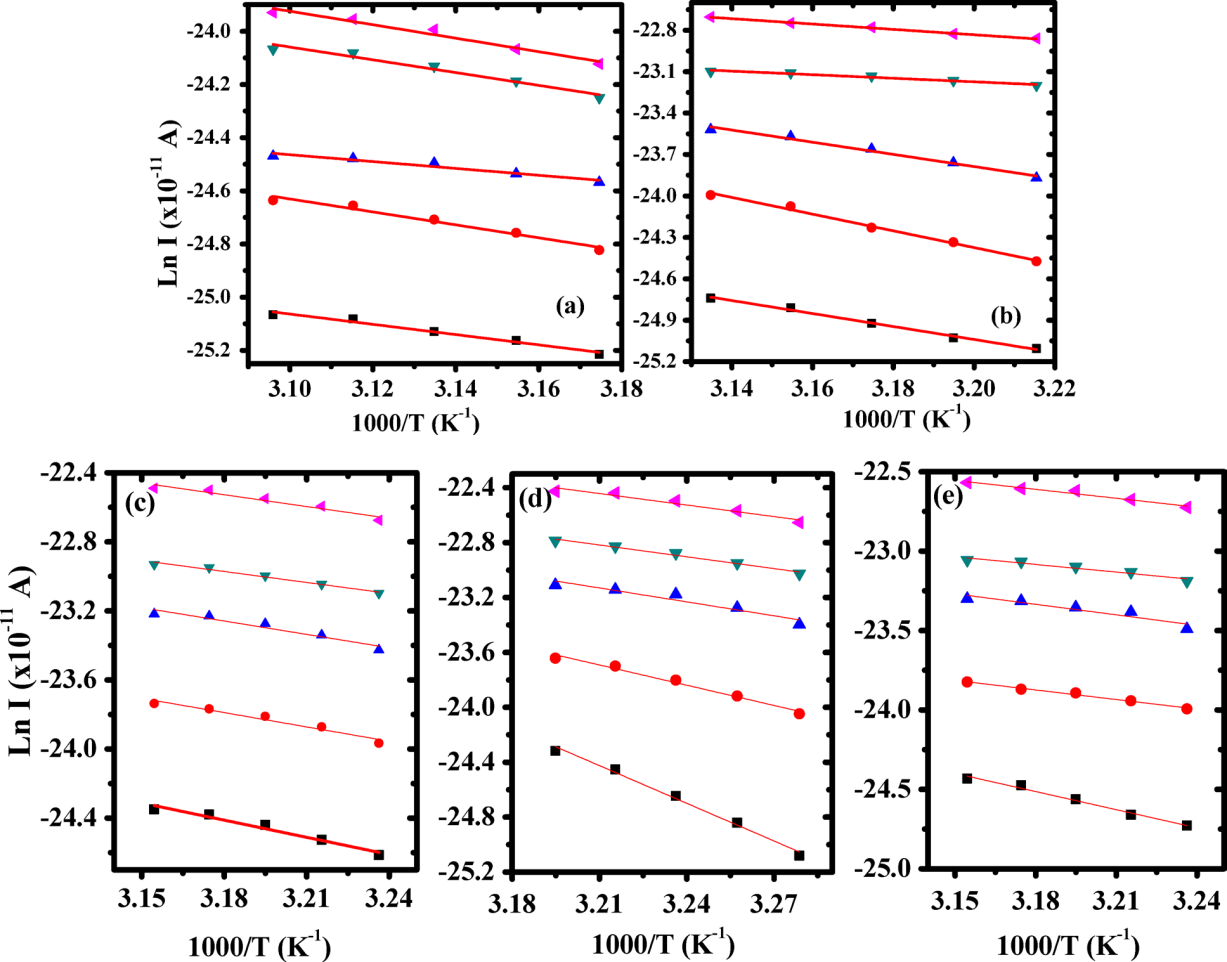
Ln I vs. 1000/T of (**a**) pure P(VDF-TrFE), (**b**) 0.04 wt%, (**c**) 0.13 wt%, (**d**) 0.28 wt% and (**e**) 0.48 wt% Ag/P(VDF-TrFE) nanocomposite at different values of E_p_. )1 × 10^6^ V/m )3 × 10^6^ V/m )5 × 10^6^ V/m )7 × 10^6^ V/m ) 1 × 10^7^ V/m.

Values of activation energy (*E*_*a*_) associated with the TSDC relaxation peak of both P(VDF-TrFE) and Ag/P(VDF-TrFE) nanocomposites were calculated using initial rise method, i.e., by plotting Ln I versus 1/T, as depicted in Fig. 13. The values of (*τ*_*o*_) are also estimated as follows^77^.

8 $$\tau _{0} = \frac{{k_{B} T^{2} \max }}{{bE_{a} }}\exp \left( { - \frac{{E_{a} }}{{k_{B} T\max }}} \right)$$

where, *k*_*B*_, and *b* are Boltzmann’s constant and rate of heating, respectively. Molecular parameters such as, *T*_*m*_, *E*_*a*_ and *τ*_*o*_ of P(VDF-TrFE) and Ag/P(VDF-TrFE) nanocomposites are determined and summarized in Table 4.

**Table 4:** The moleculat parameters such as of PVDF-TrFE and Ag/PVDF-TrFE NCs.

Molecular parameters	E_*p*_ (10^6^ V/m)	I _max_ (10^− 11^ A)	T_max_ (K)	E_a_ (eV)	τ (sec)
PVDF-TrFE	1	1.29	223	0.17	8.8 × 10^− 2^
	3	2.02	223	0.21	7.4 × 10^− 3^
	5	2.37	223	0.11	2.38
	7	3.55	223	0.21	8.6 × 10^− 3^
	10	4.04	223	0.22	4.5 × 10^− 3^
0.04wt % Ag/ PVDF-TrFE NC	1	1.96	321	0.41	1.9 × 10^− 4^
	3	4.02	321	0.52	2.1 × 10^− 6^
	5	6.35	321	0.38	5 × 10^− 4^
	7	9.5	321	0.11	28.8
	10	14	321	0.17	2.48
0.13wt % Ag/ PVDF-TrFE NC	1	2.73	319	0.29	1.8 × 10^− 2^
	3	5.15	319	0.24	0.12
	5	8.37	319	0.22	0.23
	7	11.16	319	0.18	1.23
	10	17.5	319	0.20	0.7
0.28wt % Ag/ PVDF-TrFE NC	1	3.24	317	0.79	6,3 × 10^− 11^
	3	5.88	317	0.42	7.49 × 10^− 5^
	5	9	317	0.29	1.37 × 10^− 2^
	7	13.08	317	0.25	8.29 × 10^− 2^
	10	19	317	0.24	0.1
0.48wt % Ag/ PVDF-TrFE NC	1	2.45	320	0.33	3.64 × 10^− 3^
	3	4.5	320	0.17	1.89
	5	7.9	320	0.19	0.92
	7	10.14	320	0.14	8.22
	10	16.05	320	0.16	3.1

### Piezoelectric activity

**Fig. 14:**
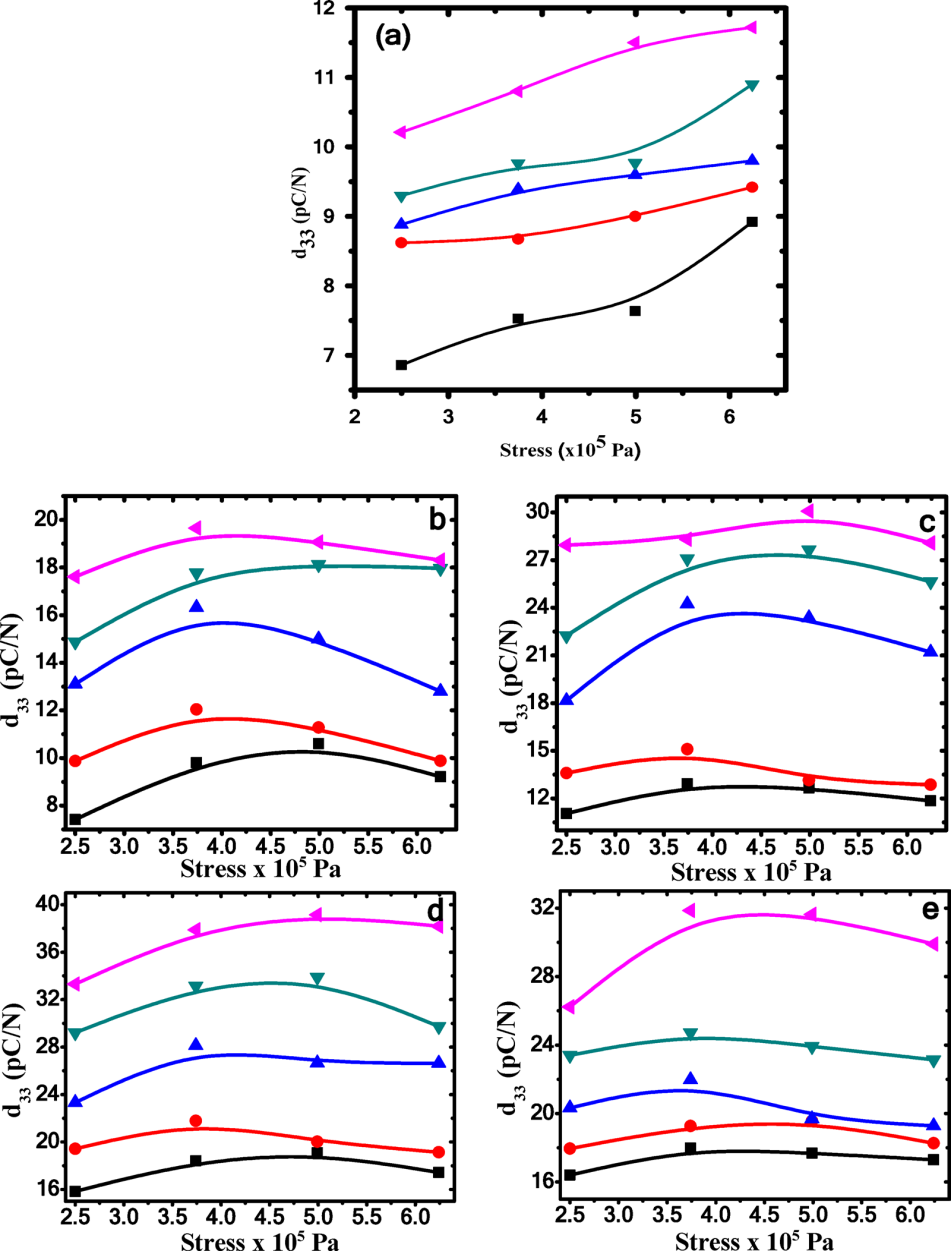
The piezoelectric coefficient d_33_ against the applied stress for (**a**) pure P(VDF-TrFE), (**b**) 0.04wt.% AgNPs, (**c**) 0.13wt.% AgNPs, (**d**) 0.28 wt% AgNPs and (**e**) 0.48 wt% AgNPs. at different measuring temperature )T_m_= 323 K ) T_m_= 333 K ) T_m_= 343 K ) T_m_= 353 K ) T_m_= 363 K.

Figure 14 illustrates the effect of the applied stress on the piezoelectric coefficient (d_33_) at various measuring temperatures for both P(VDF-TrFE) and Ag/P(VDF-TrFE) nanocomposite samples. The piezoelectric coefficient d_33_ is calculated using the following equation^78^:

9 $$\:{d}_{33}=\frac{{Q}_{3}}{A}\:\frac{{A}^{o}}{F}$$

where Q_3_ is the charge in z-direction, A^o^ is the area of applied stress, A is the electrode area and F is the force in z-direction. It has been noted that the piezoelectric coefficient d_33_ exhibits a nonlinear increase as the applied stress is augmented. Based on the dimensional effect model, applying external stress can readily alter the distance between polymer chains. Consequently, the externally applied stress has a significant impact on the value of d_33_ coefficient^78^. Additionally, the application of pressure on the samples leads to more dipoles being oriented upward, which in turn enhances the polarization of the dipoles in the sample, denoted as ΔP, and creates an effective electric field within the sample plane. This electromechanical interaction essentially results in a substantial piezoelectric effect^49^. Moreover, it has been observed that d_33_ values for all samples increase with rising measuring temperatures (T_m_). As the temperature rises, the movement of molecular chains will become more pronounced, causing the dipole moments to align more effectively with the external electric field, thereby improving the piezoelectric effect^79^.

**Fig. 15:**
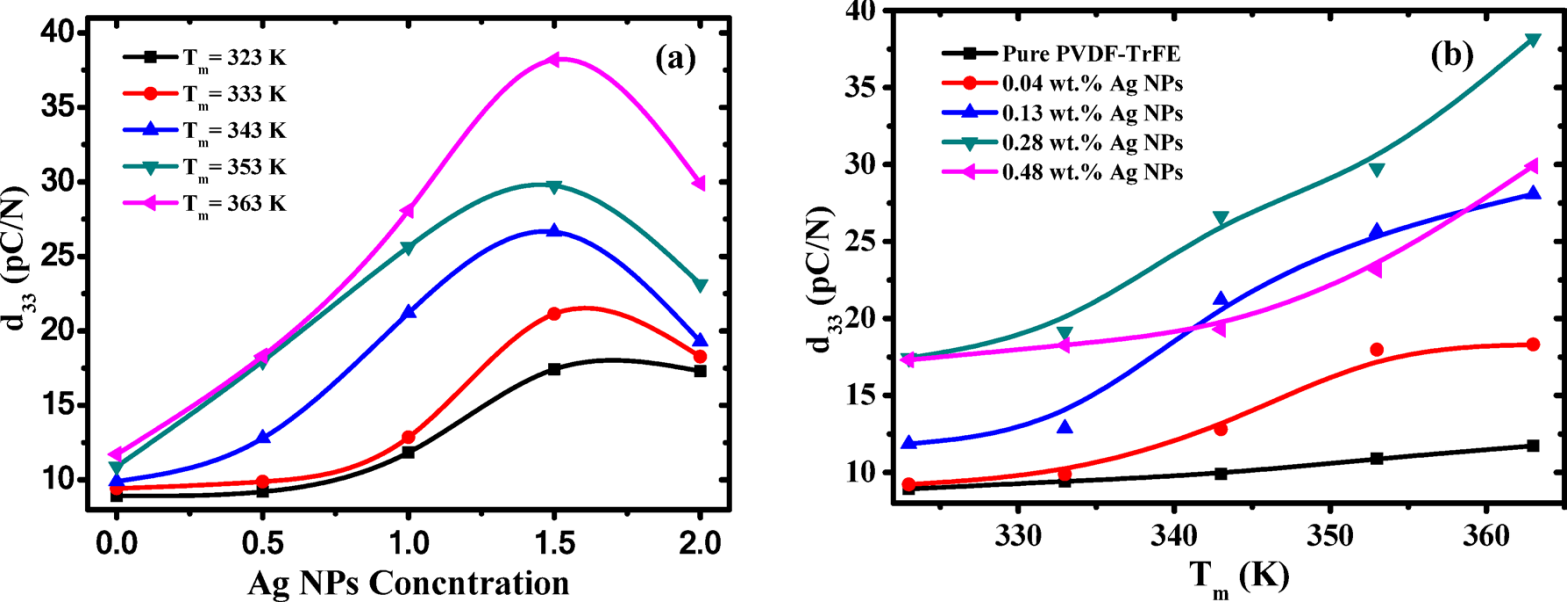
(**a**) The piezoelectric coefficient d_33_ against Ag NPs (wt%) at constant stress and various measuring temperature T_m_ and (**b**) The piezoelectric coefficient d_33_ against the measuring temperature T_m_ and constant pressure at 6.24 × 10^5^ Pa for pure P(VDF-TrFE) and different concentrations of AgNPs (wt%).

Figure 15a shows the behavior of piezoelectric coefficient versus AgNPs concentration at different values of measuring temperature (T_m_) under a constant applied stressof 6.24 × 10^5^ Pa. The (d_33_) was found to increase nonlinearly to a maximum value for 0.28 wt% of AgNPs and then decrease. This behavior of d_33_ may be due to ferroelectric domains saturation at this concentration^66^. It is worth noting that the piezoelectric response in nanocomposite samples arises from the reversible transformation of the overall distorted β-phase structure of the -CH_2_−/−CF_2_− dipoles exist in the matrix of P(VDF-TrFE) to more stable configuration, and vice versa upon application of an external stress. The charges created by the incorporation of silver nanoparticles actively interact with the -CH_2_−/−CF_2_− dipole of P(VDF-TrFE) to form the nucleus of the piezoelectric β-phase via filler-induced polarization, resulting in self-polarizing samples under the influence of local stress^80^. The increased defects in the polymer chain of P(VDF-TrFE) after the inclusion of AgNPs will enhance the polarity within the sample, thus maximizing the piezoelectric response. Figure 15b illustrates the dependence of (d_33_) of P(VDF-TrFE) and Ag/P(VDF-TrFE) nanocomposite samples on the measuring temperatures at constant pressure of 6.24 × 10^5^ Pa. It is observed that the d_33_ of pure P(VDF-TrFE) is increased from 11.7 pC/N to be 38.3 pC/N for 0.28 wt%Ag/PVDF-rFE nanocomposite sample at 6.24 × 10^5^ Pa. Hence, from the above, it can be concluded that increasing the content of silver nanoparticles plays an effective role in increasing the electroactive β-phase in the nanocomposite samples and consequently enhancing the piezoelectric response.

Parangusan et al. investigated the piezoelectric activity of PVDF-HFP/Ni-ZnO nanocomposites and they found that the value of piezoelectric coefficient (d_33_) is enhanced from 0.8 pC/N for pure PVDF-HFP to 20 pC/N for PVDF-HFP/0.5 wt% Ni–ZnO and then decreased to 1.2 pC/N for PVDF-HFP/2 wt% Ni–ZnO nanocomposite^81^. Yaseen et al. reported that the value piezoelectric coefficient (d_33_) of pure P(VDF-TrFE) is enhanced from 55 pC/N to 98 pC/N for P(VDF-TrFE)/0.002 wt%rGO nanocomposite and then decreased to 39.6 pC/N for P(VDF-TrFE)/0.04 wt%rGO nanocomposite^82^. Hassan et al. published that the piezoelectric coefficient (d_33_) of pure P(VDF-TrFE) is enhanced from 9.94 pC/N to 19.03 pC/N for P(VDF-TrFE)/1.25 wt% ZnO nanocomposite and Sarhan et al. reported that that the piezoelectric coefficient (d_33_) of pure PVVH/P(VDF-TrFE) polymer blend is enhanced from 12.8 pC/N to 23.7 pC/N for PVVH/P(VDF-TrFE)/1 wt% ZnO nanocomposite^66,83^. Yang et al. studied the piezoelectric enhancement in P(VDF-TrFE) copolymer films via controlled and template-induced epitaxy, and showed that these composites have averaged d_33_ piezoelectric coefficient of 58.2 pC/N^84^.

## Conclusion

XRD and FTIR measurements pronounced an improvement in the degree of crystallinity and electroactive β-phase of P(VDF-TrFE) copolymer upon the addition of AgNPs. The reduction in both (*E*_*ig*_*/E*_*dg*_) from (2.85/4.89) for P(VDF-TrFE) to (2.19/4.13) eV for 0.28 wt% Ag/P(VDF-TrFE) nanocomposite is due to generation of new localized states in the forbidden energy gap region. The global TSDC spectra of P(VDF-TrFE) and Ag/P(VDF-TrFE) nanocomposites exhibited two various relaxation modes. The first, which accounts for the ferroelectric-paraelectric phase transition in the low-temperature range, is called dipole relaxation, while the second occurs in the temperature range of 360–400 K and is called space charge relaxation. Our measurements also revealed that the piezoelectricity of P(VDF-TrFE) composites was enhanced with increasing applied stress and silver nanoparticle content. This behavior was attributed to the reorientation of dipoles induced by the applied stress, which increases the self-polarization of the samples, as well as to the interfacial interaction between charges generated by the presence of silver nanoparticles and the dipoles. The piezoelectric coefficient (d_33_) is found to enhanced and increased from 11.7 pC/N for P(VDF-TrFE) to 38.3 pC/N for Ag/P(VDF-TrFE) nanocomposite sample at 6.24 × 10^5^ Pa. Hence, we can conclude that Ag/P(VDF-TrFE) nanocomposites give a novel structure applicable to a variety of flexible electrical and electronic applications, such as sensors, nanogenerators and energy storage and sources.

## Data Availability

The datasets analyzed during the current study available from the corresponding author on reasonable request.
